# MRI of symptomatic and asymptomatic full-thickness rotator cuff tears: A comparison of findings in 100 subjects

**DOI:** 10.3109/17453674.2010.483993

**Published:** 2010-05-21

**Authors:** Stefan Moosmayer, Rana Tariq, Morten G Stiris, Hans-Jørgen Smith

**Affiliations:** ^1^Department of Orthopaedic Surgery, Martina Hansen’s Hospital, Baerum; ^2^Unilab Diagnostics, Oslo; ^3^Department of Radiology, Oslo University Hospital, Aker, Oslo; ^4^Department of Radiology, Oslo University Hospital, Rikshospitalet, Oslo; ^5^Faculty Division Rikshospitalet, University of OsloNorway

## Abstract

**Background and purpose:**

Why some full-thickness rotator cuff tears are symptomatic and others are asymptomatic is not understood. By comparing MRI findings in symptomatic and asymptomatic tears, we wanted to identify any tear characteristics that differed between groups.

**Patients and methods:**

50 subjects with asymptomatic and 50 subjects with symptomatic full-thickness tears were examined by MRI. Tear characteristics including tear size, tear location, the condition of the long head of the biceps, atrophy, and fatty degeneration of the muscles were compared between groups.

**Results:**

Single factor logistic regression analysis showed that there were statistically significant associations between symptoms and tear size exceeding 3 cm in the medial-lateral plane, positive tangent sign, and fatty degeneration exceeding grade 1 of the supraspinatus and infraspinatus muscles.

**Interpretation:**

We found associations between the symptomatic status of a rotator cuff tear and MRI-derived tear characteristics. The causal relationships are unclear.

## Introduction

Asymptomatic full-thickness tears of the rotator cuff have been detected in sonographic (Tempelhof et al. 1999, Worland et al. 2003, Schibany et al. 2004, Moosmayer et al. 2009) and MRI screening studies (Sher et al. 1995) of individuals with no shoulder problems, and in cadaver studies (Cotton and Rideout 1964, Ozaki et al. 1988). Reported prevalences vary from 6% to 34%, and increase with age. Why these tears are asymptomatic while others cause shoulder pain and dysfunction is not understood. Differences in tear anatomy and shoulder kinematics may be suspected, but have not been detected to date. Few comparative studies have been performed, and on small patient groups only (Yamaguchi et al. 2000, Hirano et al. 2006). Based on clinical judgement, we selected MRI-derivable factors with potential importance for tear symptomatics. MRI is widely accepted as a diagnostic tool in imaging of the shoulder. It has shown high diagnostic validity for the detection of full-thickness rotator cuff tears (Ianotti et al. 1991, Teefey et al. 2004) and for the description of tear characteristics such as tear size and location (Bryant et al. 2002, Kluger et al. 2003), muscle atrophy, fatty degeneration (Thomazeau et al. 1996, Zanetti et al. 1998a, Fuchs et al. 1999), and involvement of the biceps tendon (Zanetti et al. 1998b). Our hypothesis was that morphological tear characteristics would differ between patients with symptomatic and asymptomatic tears, and that these differences would be detectable by MRI. Such information might lead to a better understanding of factors that are important in the development of symptoms in full-thickness tears of the rotator cuff.

## Patients and methods

Our study protocol was approved by our regional health ethics board (no. 288-05082) and informed consent was obtained from study subjects before examination. From September 2005 through January 2008, 50 shoulders with asymptomatic and 50 shoulders with symptomatic full-thickness tears of the rotator cuff were included. Criteria for inclusion into the asymptomatic group consisted of a history involving no earlier or acute shoulder pain, normal shoulder function, normal physical examination findings, a result of at least 90 points in the self-report section of the American Shoulder and Elbow Surgeons form (ASES) (Richards et al. 1994), and a full-thickness rotator cuff tear demonstrated at both sonography and MRI. The choice of a cut-off point of 90 on the ASES scale was based on age-related baseline values from studies of individuals with no history of shoulder problems (Sallay and Reed 2003, Thomas et al. 2003). In subjects presenting with bilateral asymptomatic tears, only one shoulder was included in the study.

Thirty individuals who fulfilled our inclusion criteria regarding at least one shoulder were found during a study of the prevalence of asymptomatic rotator cuff tears (Moosmayer et al. 2009). In this study, clinical and sonographic screening supplemented with MRI was performed on asymptomatic subjects who had been treated earlier at our hospital for minor orthopedic conditions unrelated to the shoulder. The remaining 20 asymptomatic tears were found among present outpatients referred to our hospital for a history of earlier or acute unilateral shoulder pain, and where bilateral clinical, sonographic, and MR examination showed an asymptomatic rotator cuff tear in the pain-free contralateral shoulder.

Consequently, subjects in the asymptomatic tear group differed with respect to the condition of the contralateral shoulder, and comparison between the asymptomatic and the symptomatic tear groups was supplemented by comparing each of the two asymptomatic subgroups (with or without pain contralaterally) to the symptomatic tear group.

Criteria of inclusion into the symptomatic tear group consisted of a history of atraumatic shoulder pain, physical examination findings typical of a rotator cuff tear, and a full-thickness tear as detected by sonography and MRI. In patients with bilateral rotator cuff tears, only one of the shoulders was used for study analysis. Patients with other local or systemic diseases affecting shoulder function were excluded. 50 patients who were referred to our outpatient clinic and who fulfilled our criteria were included consecutively.

Both clinical examination and sonography at baseline in both groups were performed by one examiner (SM). Details of the sonographic examination have been reported (Moosmayer et al. 2007). Range of motion (ROM) measurement for abduction, flexion, and external rotation was performed with a goniometer. Strength was assessed in kg with a handheld spring balance in 90° shoulder abduction and flexion. The “break test method” was used, in which the subject resisted the examiner’s downward directed force until the examiner overcame the subject’s isometric contraction. The average of 2 consecutive measurements was used for data analysis. The ASES score was completed by all subjects.

All MR examinations were performed on a 1.5 T scanner (Siemens Medical Systems, Erlangen, Germany). A dedicated shoulder array coil was used. The arm was placed at the side of the body with the thumb pointing upwards. The following 5 sequences, all with a slice thickness of 3.5–4 mm, a 17.5- to 18.0-cm field of view (FOV), and one number of excitations (NEX) were obtained: (1) oblique sagittal T1-weighted spin echo (TR/TE = 513/13 ms, matrix 192 × 256), (2) oblique sagittal T2-weighted turbo spin echo (TSE) (TR/TE = 2930/74 ms, matrix 218 × 256), (3) oblique coronal and (4) axial proton density-weighted TSE with fat saturation (TR/TE = 2800/40 ms, matrix 230 × 256), and (5) oblique coronal dual echo TSE (TR/TE = 2,500/13 – 81, matrix 205 × 256). A full-thickness tear was diagnosed in case of a discontinuity or a gap in the tendon or increased signal intensity (isointense compared to fluid) on T2-weighted images, extending from the articular to the bursal surface of the tendon (Ianotti et al. 1991).

MRI data were stored on a picture-archiving and communication system (PACS) workstation and the provider’s image analysis software was used for review of images. Studies from asymptomatic and symptomatic rotator cuff tears were randomly mixed and independently evaluated by 2 experienced musculoskeletal radiologists (RT and MGS). The radiologists were blinded regarding previous clinical and radiological findings. Differing results were discussed between the radiologists in order to reach a consensus.

Selection of MRI-derivable variables for further analysis was performed on the basis of clinical judgement and existing knowledge about their potential influence on symptoms from rotator cuff tears (Burkhart 1993, Thompson et al. 1996, Yamaguchi et al. 2001 and 2006, Kedgley et al. 2007). We hypothesized that there was a positive correlation between tear size, superior-anterior tear location, a biceps tendon tear, severe atrophy or fatty degeneration, and symptoms from a rotator cuff tear.

Maximum tear size on MRI was measured in oblique-sagittal and oblique-coronal planes. Tears were classified as being small/medium (up to 3 cm) or large/massive (exceeding 3 cm). Measurement was performed along a straight line, visualizing the distance between the margins of the tear (oblique sagittal plane) or between the margin of the tear and the lateral edge of the humeral articular surface (oblique coronal plane) (Teefey et al. 2004). Transition areas with markedly increased signal intensity compared to normal cuff tissue were included in the tear size.

The location of the tear (with tendon involved) was determined in the oblique sagittal plane as being superior-anterior (affecting the supraspinatus together with the rotator interval and/or the subscapularis) or superior-posterior (affecting the supraspinatus with or without extension into the infraspinatus).

The long head of the biceps muscle was classified as being intact or torn. A tear was diagnosed if the tendon could not be identified within the intertubercular sulcus or at any other place in the joint (Zanetti et al. 1998b).

Atrophy of the supraspinatus muscle was assessed by calculating the occupational ratio of the fossa (Thomazeau et al. 1996) and from the tangent sign (Zanetti et al. 1998a). Analyses were performed on the most lateral image of the oblique sagittal T1-weighted series, on which the scapular spine was in contact with the scapular body (Y-shaped view). Cross-sectional areas (CSAs; in cm^2^) of the supraspinatus muscle and of the supraspinatus fossa were determined by tracing the contour of each region, using the manufacturer’s image analysis software. The occupational ratio was calculated by dividing the CSA of the supraspinatus muscle by the CSA of the fossa. Tears were classified into groups with ratios below 0.4 (grade 3, indicating severe atrophy) or ratios of 0.4 and more (grade 1 or 2, indicating no or moderate atrophy).

For analysis of the tangent sign, an additional straight line was drawn from the top of the coracoid process to the top of the spine of the scapula. The tangent sign was determined as being positive (indicating severe atrophy) if the superior border of the muscle was below the tangent line, and as being negative if the muscle belly and tangent line intersected.

Assessment of fatty degeneration of the muscles was performed on the same MRI slice as the quantitative assessments, according to the classification of Goutaillier et al. (1994). Originally, this classification was described for CT scanning, but has been shown to be reproducible on MRI (Fuchs et al. 1999). Fatty degeneration of each of the muscle bellies of the supraspinatus, infraspinatus, and subscapularis was classified as low (grade 0 = no fat, or 1 = some fatty streaks) or as severe (grades 2–4). To differentiate localized fatty degeneration of the rotator cuff from generalized, constitutional muscular degeneration, the teres major muscle was used as a reference. This muscle was chosen because it could be evaluated on our reference slice for the rotator cuff muscles and it differs from the rotator cuff by its function and innervation.

### Statistics

Sample size calculation was performed for an expected low proportion of 0.15 in the asymptomatic group for tear characteristics that we assumed to be associated with symptoms. We wanted to be able to detect a 4-fold higher odds of being symptomatic (OR = 4) for subjects presenting the tear characteristics on MRI. To achieve a significance level of 0.05 and a power of 0.8, a sample size of 43 subjects in each group would be necessary.

Demographic and clinical data at inclusion were compared between groups by t-tests or Mann-Whitney U tests for continuous parameters and chi-squared tests for categorical parameters. The age of the subject at inclusion was considered to be a confounder, and all logistic regression analyses were performed while adjusting for age. Associations between MRI-derived variables (independent factors) and tear group (dependent factor) were tested by logistic regression. Single-factor analyses were performed between the main groups, and odds ratios, 95% CI, and p-values are given. Subsequently, single-factor analyses were performed separately between the 2 asymptomatic subgroups and the symptomatic tear group.

## Results

For demographic data, with the exception of mean subject age, no statistically significant baseline differences were found. Difference in mean age was 5 years (95% CI: 1.8–9.0), the higher mean age being in the asymptomatic group (Table 1). As a consequence of our selection criteria for the 2 groups, there was a statistically significant difference in clinical data between the symptomatic subjects and the asymptomatic subjects (Table 2).

**Table 1.** Demographic data at inclusion

	Symptomatic tears	Asymptomatic tears	p-value
No. of study subjects	50	50	
Age, years ^**a**^	64 (10)	69 (7.9)	0.004
Sex (M/F) ^**b**^	32/18	31/19	1.0
Affected side (R/L) ^**b**^	36/14	31/19	0.4
Tear on dominant/non-dominant side ^**b**^	35/15	30/20	0.4
Contralateral shoulder (AS/S) ^**b**^	32/18	28/22	0.3
Shoulder demanding activities in work, sports, leisure time (Y/N) ^**b**^	34/16	28/22	0.3

^**a**^ Values are mean (SD).

^**b**^ Values are given as no. of patients.

AS: asymptomatic; S: symptomatic.

**Table 2.** Clinical findings at inclusion

	Symptomatic tears	Asymptomatic tears	p-value
No. of study subjects	50	50	
ASES score, points ^**a**^	47 (14)	97 (3.3)	< 0.001
Pain free abduction, degrees ^**a**^	82 (36)	176 (11)	< 0.001
Pain free flexion, degrees ^**a**^	105 (43)	177 (5.5)	< 0.001
Pain-free external rotation, degrees ^**a**^	56 (17)	68 (11)	< 0.001
Strength in abduction, kg ^**a**^	4.0 (2.5)	6.6 (2.7)	< 0.001
Strength in flexion, kg ^**a**^	4.6 (3.0)	6.6 (2.7)	0.001
Pain, VAS ^**a**^	5.8 (1.6)	0.2 (0.4)	< 0.001

^**a**^Values are mean (SD).

Of the subjects with an asymptomatic tear in the index shoulder, 22 had a painful contralateral shoulder and constituted the first asymptomatic subgroup (subgroup 1) whereas 28 subjects had no pain contralaterally and they formed the second asymptomatic subgroup (subgroup 2).

### Primary study result (Table 3)

In single-factor, age-adjusted logistic regression analysis, findings that were significantly associated with symptoms from rotator cuff tears were (1) tear size exceeding 3 cm in the medial-lateral plane (OR = 4, meaning that subjects with rotator cuff tears exceeding 3 cm in the medial-lateral plane had 4-times higher odds of having symptoms than subjects with smaller tears), (2) positive tangent sign (OR = 3), and (3) severe degree (grade 2–4) of fatty degeneration in the supraspinatus (OR = 5) or infraspinatus (OR = 5) muscles.

**Table 3.** Distributions of independent variables between symptomatic and asymptomatic tear groups with results from single-factor, age adjusted logistic regression analysis. An OR of > 1 indicates a positive association between the potential predictor and symptoms

	Dependent variable			
Independent variable	Asymptomatic	Symptomatic	OR^**a**^ (95% CI)	p-value
Tear size, med.–lat.				
≤ 3 cm	39	29	4 (1.5–10)	0.007
> 3 cm	11	21		
Tear size, ant.–post.				
≤ 3 cm	41	40	2 (0.5–4.2)	0.5
> 3 cm	9	10		
Tear location				
Superior-posterior	39	34	2 (0.7–4.2)	0.3
Superior-anterior	11	16		
Biceps tendon				
No tear	41	41	1 (0.4–3.5)	0.7
Tear	9	9		
Muscle atrophy^**b**^				
Grade 1 or 2	36	31	2 (0.9–5.5)	0.09
Grade 3	14	19		
Muscle atrophy (tangent sign)				
Negative	42	36	3 (1.1–9.0)	0.04
Positive	8	14		
Fatty atrophy SSP^**c**^				
Grade 0–1	41	31	5 (1.6–13)	0.004
Grade 2–4	9	19		
Fatty atrophy ISP^**c**^				
Grade 0–1	44	35	5 (1.5–14)	0.009
Grade 2–4	6	15		
Fatty atrophy SSC^**c**^				
Grade 0–1	45	41	2 (0.6–7.3)	0.2
Grade 2–4	5	9		

^**a**^ age-adjusted odds ratios.

^**b**^ grading according to Thomazeau et al. (1996).

^**c**^ grading according to Goutallier et al. (1994).

CI: confidence interval;

SSP: supraspinatus muscle;

ISP: infraspinatus muscle;

SSC: subscapularis muscle.

In all subjects with fatty degeneration of grade 2–4 of the supraspinatus or infraspinatus muscles, the teres major muscle (our reference muscle) appeared normal on MRI (grade 0–1), indicating a localized degeneration of the affected rotator cuff muscles only.

### Secondary study result (Table 4)

We found larger ORs for all MRI-derived factors for the comparison between asymptomatic tear subgroup 2 and the symptomatic tear group. This indicates that asymptomatic tears which are accompanied by a pain-free contralateral shoulder differ more from symptomatic tears than asymptomatic tears which are accompanied by a painful contralateral shoulder.

**Table 4.** Results from single-factor, age-adjusted logistic regression analysis from the comparison between asymptomatic tear subgroups 1 and 2 and the symptomatic tear group. An OR of > 1 indicates a positive association between the potential predictor and symptoms. Values are OR ^c^ (95% confidence interval) and p-value

	Asymptomatic subgroup 1^**a**^ (n = 22) vs. Symptomatic tear group (n = 50)	Asymptomatic subgroup 2^**b**^ (n = 28) vs. Symptomatic tear group (n = 50)
Tear size, med.-lat.	3 (1.0–12)	5 (1.5–19)
≤ 3 cm vs. > 3 cm	0.05	0.008
Tear size, ant.-post.	1 (0.4–5.0)	2 (0.5–5.8)
≤ 3 cm vs. > 3 cm	0.6	0.5
Tear location,	1 (0.4–3.9)	2 (0.7–7.7)
sup.-ant. vs. sup.-post.	0.7	0.2
Biceps tendon tear,	1 (0.2–2.2)	3 (0.6–11)
tear vs. no tear	0.5	0.2
Muscle atrophy**^d^**	1 (0.4–3.3)	5 (1.4–17)
≤ vs. > grade 2	0.8	0.01
Muscle atrophy	1 (0.4–4.5)	13 (2.3–74)
pos. vs. neg. tangent sign	0.6	0.004
Fatty atrophy SSP**^e^**	2 (0.6–5.9)	24 (3.9–143)
≤ vs. > grade 1	0.3	0.001
Fatty atrophy ISP**^e^**	4 (0.9–15)	5 (1.3–23)
≤ vs. > grade 1	0.08	0.02
Fatty atrophy SSC**^e^**	2 (0.4–12)	3 (0.6–12)
≤ vs. > grade 1	0.4	0.2

^**a**^ asymptomatic tear in the index shoulder, pain in the contralateral shoulder.

^**b**^ asymptomatic tear in the index shoulder, no pain in the contralateral shoulder.

^**c**^ age-adjusted odds ratio.

^**d**^ grading according to Thomazeau et al. (1996).

^**e**^ grading according to Goutallier et al. (1994). SSP: supraspinatus muscle; ISP: infraspinatus muscle; SSC: subscapularis muscle.

## Discussion

In contrast to earlier publications, we found associations between the symptoms of a rotator cuff tear and the characteristics of the tear. This may be explained by differences in study populations, study protocols, and examination techniques. Yamaguchi et al. (2000) used scapular plane radiographs to compare humeral kinematics during active arm elevation between normal volunteers, patients with symptomatic rotator cuff tears, and subjects with asymptomatic tears. They found increased superior translation of the humeral head on the glenoid in both tear groups compared to the no-tear group, but there were no statistically significant differences between the symptomatic tear group and the asymptomatic tear group. This may have been a consequence of having a sample size of only 10 subjects in each group.

Hirano et al. (2006) used indirect MR arthrography to compare tear size and the amount of subacrominal-subdeltoid bursal fluid in 15 asymptomatic and 23 symptomatic rotator cuff tears. The findings were similar in the two groups, but the majority of the study population had partial-thickness tears and analysis of muscle atrophy and degeneration was not part of the study protocol.

In our study, we selected tear characteristics as demonstrated on MRI for analysis. On the basis of existing knowledge, we considered these characteristics to be potential predictors of the symptomatic status of rotator cuff tears. Tear size was included according to the findings of Yamaguchi et al. (2001) who, in a longitudinal study, followed asymptomatic tears sonographically and clinically over 5 years. Symptoms developed in 23 of 45 cases and there was progression of tear size in 9 of 23 cases. Of those who became symptomatic, 7 of 14 showed tear size progression, compared to 2 of 9 in the group that remained asymptomatic. In a recent cross-sectional study, Yamaguchi et al. (2006) examined tear sizes by sonography in 82 patients with a symptomatic rotator cuff tear in one shoulder and an asymptomatic tear in the other. He found larger tear sizes in the symptomatic shoulders, with a mean difference between groups of 5.4 mm. Our finding of a positive association between tear size in the medial-lateral plane and symptoms of a rotator cuff tear is therefore in good agreement with the findings of Yamaguchi et al. (2001, 2006).

We selected atrophy and fatty degeneration of the rotator cuff muscles as potential predictors of pain from a rotator cuff tear, on the basis of the idea of an anatomically deficient but functionally sound rotator cuff, as described by Burkhart (1993). According to a suspension bridge model, tears located within the area defined by the rotator cable (crescent area) might be less at risk of development of serious muscle dystrophy as force transfer from the muscles to the greater tuberosity will be possible through the cable. Consequently, tears of the supraspinatus and parts of the infraspinatus that are bypassed by an intact cable will have a lower risk of development of muscle atrophy and fatty degeneration. These muscles will still contribute as humeral depressors, and thus prevent impingement and pain. Our findings of lower frequencies of serious supra- and infraspinatus muscle atrophy and fatty degeneration in the asymptomatic tear group support this hypothesis.

The pathological long head of the biceps muscle has been proposed as a source of pain in patients with a rotator cuff tear (Szabó et al. 2008). We classified the biceps tendon only into torn or not torn, and based on this classification we found no differences between the groups. Pain from the biceps tendon is usually related to conditions such as tendonitis, delamination, subluxation, or dislocation, but these conditions were not registered in our study.

Both in the symptomatic tear group and the asymptomatic tear group, only one shoulder per subject was included. In subjects where both shoulders fulfilled our criteria for inclusion, the shoulder that (by chance) was examined first was included in the study. This was done to perform statistical analyses on the basis of independent observations.

We identified subject age as a confounder and all regression analyses were performed while controlling for age. This had to be done because subject age was associated with independent factors (older subjects had larger tear sizes, higher degrees of atrophy, and fatty degeneration) and with the dependent factor, and, at the same time, differed at baseline between groups (with higher mean age in the asymptomatic group). Not controlling for age would have led to confounding bias, and would have masked the positive associations found in this study.

Even though all tears included in the asymptomatic group represented true asymptomatic tears according to our inclusion criteria, and should thus reflect typical characteristics of the condition, they differed with respect to the status of the contralateral shoulder. One might argue that by comparing their two shoulders, subjects with pain contralaterally might tend to overlook minor symptoms in their index shoulder, as they experience this shoulder as being much better than the reference shoulder. This argument may be supported by our findings from subgroup analyses. Greater differences, as expressed by ORs, were found for MRI-derived tear characteristics between the symptomatic tear group and the subgroup with an asymptomatic tear in the index shoulder together with a pain-free contralateral shoulder. This is important, as existing evidence in the field is partly based on results from studies exclusively involving subjects with pain in the contralateral shoulder (Yamaguchi et al. 2001, 2006).

Despite the associations we found between MRI-derived tear characteristics and tear symptoms, the clinical value of these findings is limited. The cross-sectional design of our study does not permit establishment of any causal relationships between independent and dependent variables. On the other hand, our findings fit well with existing knowledge about the negative effect of increasing tear size and decreasing muscle quality on physiological shoulder kinematics (Thompson et al. 1996, Kedgley et al. 2007). Our results should mainly be used as a basis for future research. The question of whether the positive associations found in our study reflect a causal relationship should be investigated in longitudinal studies.
